# Rigid Probe Trabeculotomy Versus 360-Degree Catheter Trabeculotomy in Childhood Glaucoma

**DOI:** 10.3390/jcm13237341

**Published:** 2024-12-02

**Authors:** Felix Mathias Wagner, Paul Oster, Julia Veran Stingl, Alexander Karl-Georg Schuster, Jasmin Rezapour, Angi Liz Mendoza-Moreira, Achim Fieß, Anke Messerschmidt-Roth, Franz Grehn, Norbert Pfeiffer, Esther Maria Hoffmann

**Affiliations:** 1 Department of Ophthalmology, University Medical Center of the Johannes Gutenberg University Mainz, 55131 Mainz, Germany; felix.wagner@unimedizin-mainz.de (F.M.W.);; 2 Ophthalmology Department, Philipps University of Marburg, 35043 Marburg, Germany

**Keywords:** childhood glaucoma, trabeculotomy, glaucoma surgery

## Abstract

**Background/Objectives**: This study aims to compare the effectiveness of traditional rigid probe trabeculotomy and 360-degree catheter trabeculotomy in treating childhood glaucoma, underlining the necessity of early surgical intervention. **Methods:** This retrospective cohort study, conducted at the University Eye Hospital Mainz, Germany, included 109 patients under 18 years with childhood glaucoma who underwent rigid probe trabeculotomy or 360-degree catheter trabeculotomy between January 2015 and February 2021. **Results:** A total of 151 eyes from 109 patients were included. The average IOP decreased significantly in both groups, with a greater reduction seen in the 360-degree catheter trabeculotomy group (mean reduction: 10.1 ± 8.7 mmHg; *p* < 0.001). In the rigid probe trabeculotomy group, the IOP reduction was 8.1 ± 9.0 mmHg (*p* < 0.001). The need for revision surgeries was lower in the 360-degree catheter trabeculotomy group. **Conclusions:** Both trabeculotomy techniques effectively reduced the intraocular pressure in childhood glaucoma. The 360-degree catheter trabeculotomy group demonstrated fewer revision surgeries compared to the rigid probe trabeculotomy group. However, there was no statistically significant difference in the IOP reduction between the groups. These findings indicate that while both methods are effective in managing the IOP in childhood glaucoma, the 360-degree catheter trabeculotomy may provide more favorable long-term results.

## 1. Introduction

Childhood glaucoma is composed of a heterogeneous group of diseases, which can all lead to blindness if left untreated. Damage to the eye due to elevated intraocular pressure (IOP) is characteristic and is caused by a number of conditions [1]. Therefore, early diagnosis and treatment are necessary. The Childhood Glaucoma Research Network (CGRN) describes the current classification system [1], dividing childhood glaucoma into a number of categories, of which primary congenital glaucoma (PCG) is among the most frequent [2,3]. In adults, pharmacologic therapy for the reduction of the IOP is the most common form of treatment for glaucoma. However, pharmacologic therapy alone is indicated only if a sufficient pressure reduction can be achieved to prevent the progression of glaucomatous damage. In congenital glaucoma, pharmacologic therapy is not effective in the long term. It can be used when deciding on the surgical intervention or when the IOP reduction is insufficient after surgical procedures have been performed. The diagnosis of congenital glaucoma is thus usually accompanied by an absolute indication for surgery.

Trabeculotomy and goniotomy are the mainstay and initial procedures of choice. If the desired surgical success fails to materialize, filtration surgery may be resorted to; furthermore, multiple repetitions of the procedure may be required. Cyclodestructive treatment can be performed as an intermediate therapy or as a revision surgery preceding trabeculotomy. In particularly severe cases or in cases of secondary childhood glaucoma (especially aphakic glaucoma), drainage surgery with implantation of a shunt may be required in addition to goniotomy, trabeculotomy, and filtering surgery.

In comparison to goniotomy, trabeculotomy has the advantage that it can be performed in both clear and cloudy corneas [4]. Several surgical protocols are possible, such as traditional rigid probe trabeculotomy and 360-degree catheter trabeculotomy. Common to both is that the internal wall of Schlemm’s canal, the trabecular meshwork and the embryonic tissue are transected. In probe trabeculotomy, the opening of the inner wall of Schlemm’s canal is over approximately 120 degrees, whereas 360-degree catheter trabeculotomy allows an opening of up to 360 degrees in a single procedure [5].

Only a few retrospective comparisons between both procedures for the management of childhood glaucoma exist to date, and with small study samples [6,7]. The aim of this study was thus to evaluate the post-operative outcomes of rigid probe trabeculotomy versus 360-degree catheter trabeculotomy in childhood glaucoma surgery in a large cohort.

## 2. Materials and Methods

We conducted a retrospective cohort study involving patients who had undergone rigid probe trabeculotomy or 360-degree catheter trabeculotomy due to childhood glaucoma between January 2015 and February 2021 at the University Eye Hospital Mainz, Germany. We identified 109 patients by searching an electronic surgical case register. Eligible subjects were then confirmed by manual chart review. All the data were fully pseudonymized before they were accessed. According to regional laws, the requirement for informed consent was waived by the ethics committee of the medical board of Rhineland-Palatinate. The collected characteristics included demographics and ocular characteristics, such as the glaucoma diagnosis, pre-operative intraocular pressure (measured on the day of surgery), post-operative intraocular pressure (measured at the last available follow-up visit), number of different glaucoma medications, visual acuity, axial length, number and type of revision procedures.

The choice of surgical procedure was determined collaboratively between the attending surgeon, the patients’ parents, and, where age appropriate, the children. The decision was influenced by clinical factors such as the corneal clarity and disease severity. 

### 2.1. Inclusion and Exclusion Criteria

Patients under the age of 18 years with glaucoma who had undergone either rigid probe trabeculotomy or 360-degree catheter trabeculotomy were included. Patients who did not meet these criteria were excluded.

### 2.2. Surgical Protocol

The surgeries were performed by three specialists in glaucoma surgery with extensive experience (N.P., E.H., and F.G.). The following surgical protocols were followed for all the rigid probe and 360-degree catheter trabeculotomy procedures at the University Eye Hospital Mainz, Germany:

#### 2.2.1. Rigid Probe Trabeculotomy

Corneal traction sutures are performed at the limbus with 7.0’silk. The conjunctiva is opened approximately 10 mm behind the limbus over 4–6 mm. Tenon’s capsule is then cut and cauterized and dissected anteriorly. If the size of the buphthalmic eye is very unfavorable (the sclera and cornea are extremely thinned), two transconjunctival rectus muscle traction sutures are required to expose one of the upper quadrants for surgery. A 3.5 × 3.5 mm square scleral flap of ^1^/_2_ to ^2^/_3_ of the scleral thickness is then prepared 1 mm into the clear cornea. Another 1.5 × 3 mm flap is dissected down to the end just above the ciliary muscle.

By moving this second flap anteriorly, Schlemm’s canal is reached, opened and deroofed. The ostia of Schlemm’s canal can be easily found on both sides. The canal is then irrigated with BSS through an irrigation cannula and the flow capacity into the collector canals is checked for a flush-induced color change. To protect the cornea and lens, the anterior chamber is filled with Healon (Healon GV; Abbott Laboratories Inc., Abbott Park, IL, USA).

A metal probe is then inserted into the canal and rotated into the anterior chamber on both sides, avoiding perforation of the trabecular–Descemet bridge at the site of the deep flap. During this maneuver, the limbus is stabilized externally with hummingbird forceps in the area of the probe tip. After the trabeculotomy is completed, the second flap is sutured or not at the discretion of the surgeon. If partial filtration is considered, the second flap is removed. The superficial flap is sutured with four 10/0 nylon sutures. The conjunctiva is closed with a meandering suture of 10/0 nylon at the limbus or with 8/0 or 10/0 Vicryl sutures. In general, the Tenon capsule and conjunctiva are closed separately.

#### 2.2.2. 360-Degree Catheter Trabeculotomy

In 360-degree catheter trabeculotomy, the same procedure as in probe trabeculotomy is performed until the ostia of Schlemm’s canal are opened. Schlemm’s canal is then irrigated with an irrigation cannula and the flow ability into the collector channels is checked. To protect the cornea and lens, the anterior chamber is filled with Healon (Healon GV; Abbott Laboratories Inc., Abbott Park, IL, USA) and the illuminated catheter (iTrack™ surgical system from Ellex Medical Laser, Mawson Lakes, Australia) is filled with viscoelastic. Two forceps are used to advance the catheter clockwise 360 degrees. Once the anterior end of the catheter emerges from the other ostium, it is grasped and gently pulled at the ends until the trabecular meshwork is severed in the form of a trabeculotomy. Before this maneuver, a small paracentesis is created to inject acetylcholine in case of pupillary dilation. The wound is closed in the same manner as described for rigid probe trabeculotomy [8,9].

In this study, if Schlemm’s canal is opened to an extent greater than 120 degrees but less than 300 degrees, the procedure is classified as an incomplete catheter trabeculotomy. For catheter retrieval, in cases where Schlemm’s canal is opened between 120 and 300 degrees, we perform a cut down into the sclera to release the catheter.

### 2.3. Outcome Measures

The primary outcome was the reduction of the IOP over time. The secondary outcomes were the number of revision surgeries, the axial length development and the visual acuity.

### 2.4. Statistical Analysis

The Mann–Whitney U test, Kruskal–Wallis test and Wilcoxon signed-rank test were used to compare differences between the surgical study groups, as appropriate. A random forest regression model was employed to assess the influence of pre-operative factors on the IOP reduction. Categorical variables were encoded using one-hot encoding, and missing values were imputed using the mean strategy. Separate models were trained for each surgical method. Feature importance was evaluated to identify the most influential predictors in each subgroup.

This was an explorative study and a *p*-value of 0.05 or less was considered statistically significant. Statistical analyses were carried out with SPSS, version 24.0 [10], and R, version 4.0.3 [11], and the packages ggplot2 [12], dplyr [13] and rstatix [14].

## 3. Results

We included 151 eyes of 109 patients with childhood glaucoma who had received either rigid probe trabeculotomy or 360-degree catheter trabeculotomy. The mean follow-up time was 34.4 months for the entire cohort. The subgroup-specific mean follow-up times were as follows: 360-degree catheter trabeculotomy (36.1 ± 6.8 months), incomplete 360-degree trabeculotomy (30.2 ± 7.3 months), and rigid probe trabeculotomy (33.4 ± 5.6 months). The study population consisted of 64 females (58.7%) and 45 males (41.3%), with a mean age of 4.7 ± 5.2 years. Of the 151 eyes, 79 (52.3%) had a diagnosis of congenital glaucoma; in 72 (47.7%) eyes, another form of childhood glaucoma was present. Moreover, 360-degree catheter trabeculotomy was performed in 65 eyes (43.0%), incomplete 360-degree catheter trabeculotomy was performed in 13 eyes (8.6%), and probe trabeculotomy was performed in 73 cases (48.3%). Table 1 summarizes the baseline characteristics of our study sample.

Primary Outcome: IOP development

**Figure 1:**
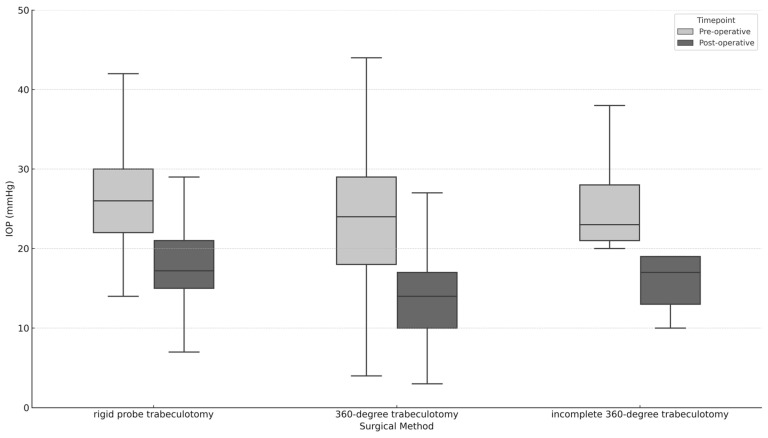
Comparison of the pre-operative IOP to the IOP at the last follow-up.

At the time of the surgery, the average intraocular pressure (IOP) was 25.1 ± 7.9 mmHg. We found no significant difference in the pre-operative IOP among the different surgery groups (*p* = 0.128). Figure 1 illustrates how the pre-operative IOP compares to the IOP at the final follow-up. By the last follow-up, the average IOP decreased to 16.4 (SD = 6.8) mmHg. Specifically, the IOP reductions for eyes initially treated with 360-degree trabeculotomy, incomplete 360-degree trabeculotomy, and rigid probe trabeculotomy were 10.1 (SD = 8.7) mmHg, 4.4 (SD = 10.9) mmHg, and 8.1 (SD = 9.0) mmHg, respectively. The reduction in the IOP was significant for both the 360-degree trabeculotomy (*p* < 0.001) and rigid probe trabeculotomy (*p* < 0.001). The reduction in the IOP for the incomplete 360-degree catheter trabeculotomy cases was 4.4 ± 10.9 mmHg, which did not reach statistical significance (*p* = 0.17). This reduction was markedly lower when directly compared to complete 360-degree trabeculotomy (*p* = 0.043) and rigid probe trabeculotomy (*p* = 0.37). There was no significant difference in the amount of IOP reduction between the different surgery groups overall (*p* = 0.14). These and other results are summarized in Table 2. There was also no significant difference in the IOP reduction between eyes with and without a history of previous surgery (*p* = 0.21).

A random forest regression model was used to predict the reduction in intraocular pressure after surgery. The model included the baseline IOP, age at surgery, sex, diagnosis, pachymetry, axial length and number of pre-operative glaucoma medications.

In the overall dataset, the random forest model explained 31% of the variance (R^2^ = 0.31), with a mean squared error (MSE) of 99.28. Feature importance analysis revealed that the baseline intraocular pressure was the most influential predictor, contributing 65.4% of the total variance explained by the model. The pachymetry (9.9%), age (9.2%), and axial length (7.7%) also contributed to the model’s predictive performance, although to a lesser extent. The number of pre-operative medications, diagnosis, history of previous operation and sex contributed minimally to the model’s variance (∑ 7.7%).

When stratifying by surgical methods, the model’s performance varied. For the rigid probe group, the model explained 37% of the variance (R^2^ = 0.37, MSE = 32.76), with the baseline IOP being the dominant factor (56.6%), followed by the pachymetry (14.2%) and age (13.5%). The axial length also played a role (10.6%), while the number of pre-operative medications, diagnosis, history of previous operation and sex contributed marginally (∑ 5.1%).

In the 360-degree trabeculotomy (360-Grad-TO) group, the model explained 54% of the variance (R^2^ = 0.54, MSE = 45.43), where the baseline intraocular pressure had an even stronger influence, accounting for 75.3% of the variance. The pachymetry (6.0%), age (5.5%) and axial length (5.5%) contributed modestly to the model’s predictive performance. In this group as well, the number of pre-operative medications, diagnosis, history of previous operation and sex contributed marginally (∑ 7.7%).

To further investigate the outcomes based on the specific type of childhood glaucoma, we performed a subgroup analysis comparing primary and secondary childhood glaucoma, as well as the effects of 360-degree trabeculotomy and rigid probe trabeculotomy, within each subgroup.

### 3.1. Primary Childhood Glaucoma

At the time of surgery, the mean intraocular pressure (IOP) for the primary childhood glaucoma group (entire cohort) was 24.04 ± 8.01 mmHg. By the final follow-up, the mean IOP had decreased to 14.67 ± 5.16 mmHg, resulting in a significant mean reduction of 9.37 ± 8.31 mmHg (*p* < 0.001).

For eyes treated with 360-degree trabeculotomy, the mean IOP at the time of surgery was 23.27 ± 7.71 mmHg. At the final follow-up, the IOP had decreased to 12.89 ± 4.50 mmHg, with a mean reduction of 10.38 ± 7.38 mmHg (*p* < 0.001). In the rigid probe trabeculotomy group, the mean pre-operative IOP was 26.34 ± 7.75 mmHg, which decreased to 16.98 ± 4.71 mmHg at the final follow-up, resulting in a mean IOP reduction of 9.36 ± 8.53 mmHg (*p* < 0.001).

When comparing the two surgical methods for primary childhood glaucoma, there was no significant difference in the pre-operative IOP (*p* = 0.23, Mann–Whitney U test) or in the IOP reduction between the two techniques at the final follow-up (*p* = 0.58, Mann–Whitney U test).

### 3.2. Secondary Childhood Glaucoma

In the secondary childhood glaucoma group, the mean pre-operative IOP for the entire cohort was 26.19 ± 7.71 mmHg. By the final follow-up, the mean IOP had decreased to 18.24 ± 7.86 mmHg, with a significant mean reduction of 7.95 ± 9.90 mmHg (*p* < 0.001).

For eyes treated with 360-degree trabeculotomy, the mean pre-operative IOP was 24.57 ± 10.01 mmHg, which decreased to 14.96 ± 5.09 mmHg at the final follow-up. The mean IOP reduction was 9.60 ± 10.92 mmHg (*p* < 0.001). For rigid probe trabeculotomy, the mean pre-operative IOP was 26.83 ± 6.50 mmHg, which decreased to 19.50 ± 8.40 mmHg at the final follow-up, resulting in a mean IOP reduction of 7.34 ± 9.28 mmHg (*p* < 0.001).

There was no significant difference in the pre-operative IOP between the two surgical methods (*p* = 0.16, Mann–Whitney U test) or in the amount of IOP reduction between the two techniques at the final follow-up (*p* = 0.58, Mann–Whitney U test).

### 3.3. Revision Surgeries

Forty-seven (31.1%) eyes required at least one revision surgery. Of the 65 eyes which were operated on using the 360-degree trabeculotomy method, 4 eyes (6.2%) required at least one revision surgery. Of the 13 eyes that received an incomplete 360-degree trabeculotomy, 3 eyes (23.1%) required at least one revision surgery. Of the 73 eyes that were treated by rigid probe trabeculotomy, 40 eyes (54.8%) required at least one revision surgery. Eyes treated by means of 360-degree trabeculotomy required significantly fewer revision surgeries than those treated by means of probe trabeculotomy (*p* < 0.001).

### 3.4. Visual Acuity

At baseline, the mean visual acuity at the time of surgery was −0.6 ± 0.5 logMAR, while the median was −0.5 logMAR. The last determined visual acuity averaged at −0.5 ± 0.5 logMAR, while the median was −0.4 logMAR. There was no significant change in visual acuity over time, irrespective of the type of surgery (*p* ≥ 0.11). Equally, there were no significant difference regarding the change of visual acuity between the groups (*p* = 0.53).

### 3.5. Axial Length

At baseline, the mean axial length at the time of surgery was 23.0 ± 2.8 mm, the median was 23.1 mm, and there was no significant difference between the eyes treated by 360-degree catheter trabeculotomy compared to those treated by rigid probe trabeculotomy. The last determined axial length averaged 23.2 ± 2.5 mm, while the median was 22.9 mm. In eyes treated by 360-degree catheter trabeculotomy, there were no significant changes regarding the pre- and post-operative axial length (23.1 ± 2.1 mm to 22.9 ± 2.0 mm; *p* = 0.06) during the follow-up. However, in eyes treated by rigid probe trabeculectomy, the axis length increased significantly from a mean of 22.9 ± 3.3 mm to a mean of 23.6 ± 3.0 mm during the follow-up. Eyes treated by means of rigid probe trabeculotomy exhibited a significantly greater change in axis length than those treated by means of 360-degree trabeculotomy (*p* = 0.001).

## 4. Discussion

In this retrospective cohort study with a mean follow-up of 34.4 months (2.9 years), we observed that while all three groups, including the subset with incomplete 360-degree trabeculotomies, showed a reduction in intraocular pressure (IOP), the differences were not statistically significant. This suggests that each method—rigid probe trabeculotomy, complete 360-degree catheter trabeculotomy, and incomplete 360-degree trabeculotomy—has its merits in the management of childhood glaucoma.

However, it is noteworthy that there was a trend toward a more effective IOP reduction in the 360-degree catheter trabeculotomy group. Although this did not reach statistical significance, it could indicate a potentially more beneficial outcome with the complete 360-degree procedure. This observation warrants further investigation, as it might have implications for surgical decision-making in pediatric glaucoma cases.

The lack of significant differences in the IOP reduction between these groups suggests that both rigid probe trabeculotomy and 360-degree catheter trabeculotomy, whether complete or incomplete, are viable options for treating childhood glaucoma.

Lim ME et al. compared traditional trabeculotomy (less than 360 degrees) to 360-degree circumferential trabeculotomy in pediatric glaucoma patients. The study revealed that both methods significantly lowered the IOP in children. Notably, one year after surgery, the mean IOP was 17.05 ± 5.92 mm Hg in the traditional group and 11.0 ± 2.31 mm Hg in the 360-degree group [7].

Our findings align with previous studies on trabeculotomy techniques in pediatric glaucoma. Lim et al. reported that 360-degree catheter-assisted trabeculotomy achieved a greater reduction in the intraocular pressure (IOP) (mean post-operative IOP: 11.0 ± 2.31 mmHg) compared to traditional trabeculotomy (17.05 ± 5.92 mmHg), with a success rate of 94.4% versus 66.7% [7]. While our study observed similar trends, the differences in the IOP reduction were not statistically significant, likely due to differences in patient demographics, sample sizes, and the longer follow-up in our cohort, which included more secondary glaucoma cases. Khalil demonstrated an adjusted trabeculotomy outcome with mean IOP reductions from 23.4 ± 8.8 mmHg to 11.5 ± 3.5 mmHg at one year (44.2% reduction) [15].

Girkin et al. similarly found better outcomes in primary congenital glaucoma (PCG) cases, emphasizing the role of patient characteristics in surgical success [16].

Shi et al. reported IOP reductions of 47.3% for microcatheter-assisted trabeculotomy and 34.2% for rigid probe trabeculotomy, comparable to our results. Further, they observed fewer revision surgeries and lower reliance on glaucoma medications in the 360-degree group compared to rigid probe trabeculotomy [6].

El-Sayed et al. found, comparable to our results, an IOP reduction in the microcatheter-assisted group from 25.1 ± 6.4 mmHg to 11.9 ± 3.4 mmHg and from 22.3 ± 5.2 mmHg to 12.8 ± 4.4 mmHg in the probe group one year after surgery [17].

Lai et al. demonstrated a slightly higher IOP reduction, with 16.1 ± 9.1 mmHg, compared to our results, which may be attributed to the fact that their pre-operative IOP was also higher, at 30.41 ± 6.09 mmHg [18].

Dragosloveanu et al. reported similar results regarding the IOP reduction for the probe trabeculotomy (25.43 ± 2.77 mmHg to 15.88 ± 2.10 mmHg) and the 360°-trabeculotomy (26.76 ± 4.78 mmHg to 13.54 ± 1.96 mmHg). In addition, they stated fewer complications and reoperations with circumferential trabeculotomy compared to traditional methods, with the reoperation rates more than five times higher in the probe trabeculotomy group, which is analogous to the findings of our own study [19].

Consistent with Hoffmann et al., we found axial length increases post-rigid probe trabeculotomy, suggesting ongoing globe expansion, whereas 360-degree trabeculotomy demonstrated stable axial lengths [8].

The differences in patient demographics and baseline characteristics across studies highlight the variability in the outcomes of trabeculotomy techniques. El Sayed et al. focused on primary congenital and secondary pediatric glaucoma cases, with 68% consanguinity and a mean surgical age of 5.6 months in the microcatheter group and 4.4 months in the rigid probe group. By contrast, Lai et al. exclusively studied primary congenital glaucoma (PCG) with a slightly older cohort (diagnosed at 0–3 years) and a mean pre-operative IOP of 30.41 mmHg.

Khalil et al. examined juvenile glaucoma with a significantly older patient group (median 21.5 years) and a baseline IOP of 23.4 mmHg. Their study also included 90.7% of eyes with angle dysgenesis. Demirok et al. included both PCG (62.5%) and juvenile glaucoma (37.5%) patients, with a median surgical age of 8.3 years and a high baseline IOP of 36.84 mmHg.

Lim et al. compared traditional and 360-degree trabeculotomy in pediatric glaucoma, predominantly PCG (75.8%), with mean surgical ages of 1.52 and 0.61 years, respectively. Shi et al. provided insights into primary and secondary glaucoma cases.

The novelty of our study lies in its direct comparison of rigid probe and 360-degree catheter trabeculotomy within a diverse cohort over an extended follow-up period. Unlike previous research focused on PCG, our study provides insights into the outcomes across various childhood glaucoma types.

In conclusion, our study highlights the effectiveness of both trabeculotomy techniques in childhood glaucoma treatment. The extent to which one method is superior to the other remains a topic for further research. This suggests the need for more comprehensive studies to conclusively determine the most effective surgical approach for childhood glaucoma.

Our results are in line with previous studies in smaller cohorts, which assessed both surgical procedures after 12 months and also showed a significantly greater reduction in the IOP in childhood glaucoma treated by 360-degree catheter trabeculotomy [6,7]. Patients who received 360-degree trabeculotomy also necessitated less glaucoma medication after surgery. Fewer surgical procedures and fewer post-operative medications could reduce healthcare costs as well as the risk of iatrogenic complications. In some cases, the surgeon opted for rigid probe trabeculotomy due to anatomical challenges, such as extreme buphthalmos or significant Schlemm’s canal obstructions, which precluded 360-degree catheterization. While this decision was guided by clinical necessity, it may have introduced bias in the comparative analysis of the outcomes. As has been shown in previous studies, achieving 360-degree canalization is not always possible due to anatomical or pathological obstructions. In our cohort, 13 eyes required incomplete 360-degree catheter trabeculotomy due to intraoperative limitations. This subgroup demonstrated a less effective IOP reduction compared to complete canalization. These outcomes highlight the need for pre-operative risk assessment and intraoperative strategies to optimize canalization success [6,7,16,20].

The retrospective study design has several limitations. The follow-up examinations were not standardized, and the intervals varied between patients, limiting the comparability of time-dependent outcomes. The study population was heterogeneous regarding the different forms of childhood glaucoma, and the absence of a standardized risk stratification system for assessing case complexity may have introduced bias into the outcome comparisons. Furthermore, treatment selection bias cannot be excluded, as the choice of procedure was influenced by clinical considerations rather than random allocation.

In conclusion, both 360-degree and rigid probe trabeculotomy significantly reduced the IOP in children with pediatric glaucoma. Future prospective studies are necessary to show which method is superior.

## Figures and Tables

**Table 1:** Baseline characteristics.

	All Patients	360° Trabeculotomy	Probe Trabeculotomy
No. of eyes (patients)	151 (109)	65 (49)	73 (59)
Age, mean (IQR), years	4.7 (0.5–8.7)	3.9 (0.4–5.1)	5.4 (0.5–9.6)
Female sex % (n)	58.7 (64)	49.2 (32)	61.6% (45)
Glaucoma type according to CGRN [1] in % (no.) *			
Primary congenital glaucoma	52.3 (79)	66.2 (43)	41.1 (30)
Juvenile open-angle glaucoma	6.6 (10)	10.8 (7)	4.1 (3)
Glaucoma following cataract surgery	14.6 (22)	9.2 (6)	24.8 (18)
Glaucoma associated with nonacquired systemic disease or syndrome	6.6 (10)	7.7 (5)	6.8 (5)
Glaucoma associated with nonacquired ocular anomalies	16.6 (25)	3.1 (2)	19.2 (14)
Glaucoma associated with acquired conditions	3.3 (5)	3.1 (2)	4.1 (3)
Proportion of eyes with previous operations % (no) ^§^	42.2 (64)	29.2 (19)	52.1 (38)
Eye laterality, right % (no.)	52.3 (79)	49.2 (32)	54.8 (40)

IQR = interquartile range; SD = standard deviation. For reasons of clarity, the values of the 11 patients (13 eyes) in the incomplete 360° TO group are not included in the table. The specific values of this group were as follows: * primary congenital glaucoma n = 6; glaucoma associated with nonacquired ocular anomalies n = 7. ^§^ Proportion of eyes with previous operations % (no) 53.9 (7).

**Table 2:** Summary of the pre-operative and post-operative clinical outcomes by surgery type.

Baseline Characteristics of Patients			
Variable	Mean ± SD		
Intraocular Pressure (mmHg)	25.1 ± 7.9		
Visual Acuity (logMAR)	−0.6 ± 0.5		
Axial Length (mm)	23.0 ± 2.8		
**Changes in Intraocular Pressure (IOP) by Surgery Type**			
Surgery Type	Baseline IOP (mmHg) (IQR)	Change in IOP (mmHg)	*p*-value for change
360° Trabeculotomy	23.7 ± 8.5 (18.0–29.0)	10.1 ± 8.7	<0.001
Incomplete 360°-TO	23.6 ± 8.6 (21.0–28.0)	4.4 ± 10.9	0.17
Rigid Probe Trabeculotomy	26.6 ± 6.9 (22.00–30.0)	8.1 ± 9.0	<0.001
*p*-value between groups			0.15
**Axial Length (AL) Changes by Surgery Type**			
Surgery Type	PreOp AL (mm)	PostOp AL (mm)	*p*-value for change
	Mean ± SD	Mean ± SD	
360-Degree Trabeculotomy	23.1 ± 2.1	22.9 ± 2.0	0.06
Rigid Probe Trabeculotomy	22.9 ± 3.3	23.6 ± 3.0	<0.05
*p*-value between groups			0.001
**Visual Acuity Outcomes**			
Time Point	Mean ± SD (logMAR)	*p*-value for change	*p*-value between groups
Pre-operative visual acuity	−0.6 ± 0.5		
Post-operative visual acuity	−0.5 ± 0.5	≥0.11	0.53

IQR = interquartile range; SD = standard deviation; TO = trabeculotomy. The “*p*-values for change” refer to the statistical tests evaluating whether the differences between pre-operative and post-operative measures (e.g., intraocular pressure or axial length) are significant within each group. The “*p*-values between groups” assess whether the differences in outcomes (e.g., IOP reduction or axial length changes) between surgical groups are statistically significant. These *p*-values were calculated using paired t-tests or Wilcoxon tests for within-group comparisons, depending on the data distribution. For between-group comparisons, ANOVA was used when the assumptions of normality and homogeneity of variance were met; otherwise, non-parametric tests such as the Mann–Whitney U test were applied.

## Data Availability

Dataset available on request from the authors.
